# Supplementary figures and images for: The Escherichia coli serS gene promoter region overlaps with the rarA gene
Source: PLoS One. 2022 Apr 15;17(4):e0260282. doi: 10.1371/journal.pone.0260282 (PMC9012371; doi:10.1371/journal.pone.0260282)

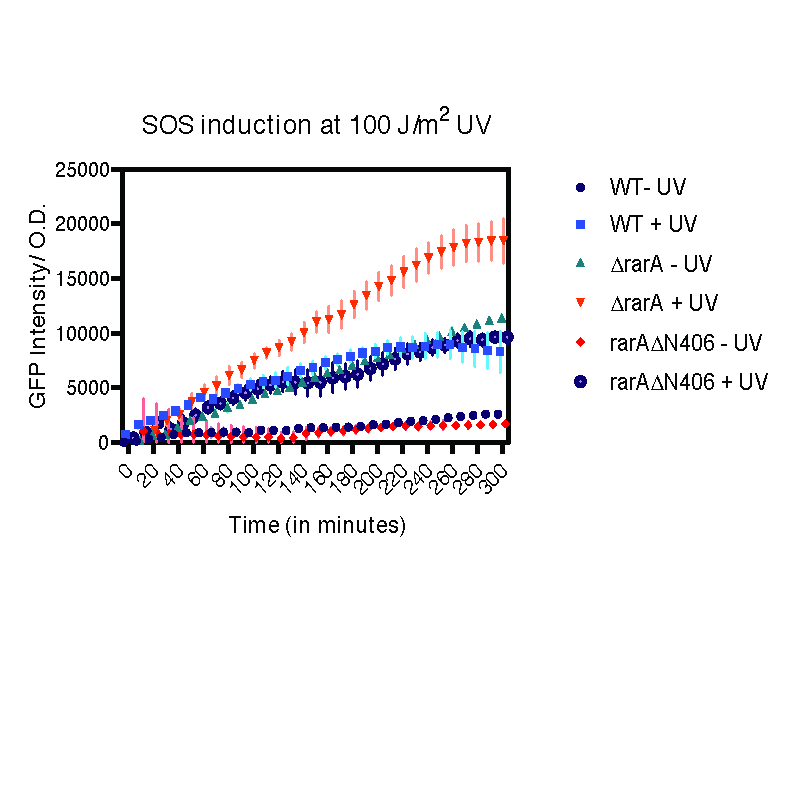

Supplement: S2 Fig — Complete deletion of rarA induces SOS response more than WT cell, in presence and absence of UV exposure. (TIFF) [file pone.0260282.s002.tiff]
