# Supplementary material for: The Escherichia coli serS gene promoter region overlaps with the rarA gene
Source: PLoS One. 2022 Apr 15;17(4):e0260282. doi: 10.1371/journal.pone.0260282 (PMC9012371; doi:10.1371/journal.pone.0260282)
Supplement: S1 File — (DOCX) [file pone.0260282.s003.docx]

**Sequences of plasmids used in this study**

**1. pEAW1012 - rarA in pRC7 plasmid**

1 caattcggga caccatcgaa tggtgcaaaa cctttcgcgg tatggcatga tagcgcccgg

61 aagagagtca attcagggtg gtgaatgtga aaccagtaac gttatacgat gtcgcagagt

121 atgccggtgt ctcttatcag accgtttccc gcgtggtgaa ccaggccagc cacgtttctg

181 cgaaaacgcg ggaaaaagtg gaagcggcga tggcggagct gaattacatt cccaaccgcg

241 tggcacaaca actggcgggc aaacagtcgt tgctgattgg cgttgccacc tccagtctgg

301 ccctgcacgc gccgtcgcaa attgtcgcgg cgattaaatc tcgcgccgat caactgggtg

361 ccagcgtggt ggtgtcgatg gtagaacgaa gcggcgtcga agcctgtaaa gcggcggtgc

421 acaatcttct cgcgcaacgc gtcagtgggc tgatcattaa ctatccgctg gatgaccagg

481 atgccattgc tgtggaagct gcctgcacta atgttccggc gttatttctt gatgtctctg

541 accagacacc catcaacagt attattttct cccatgaaga cggtacgcga ctgggcgtgg

601 agcatctggt cgcattgggt caccagcaaa tcgcgctgtt agcgggccca ttaagttctg

661 tctcggcgcg tctgcgtctg gctggctggc ataaatatct cactcgcaat caaattcagc

721 cgatagcgga acgggaaggc gactggagtg ccatgtccgg ttttcaacaa accatgcaaa

781 tgctgaatga gggcatcgtt cccactgcga tgctggttgc caacgatcag atggcgctgg

841 gcgcaatgcg cgccattacc gagtccgggc tgcgcgttgg tgcggatatc tcggtagtgg

901 gatacgacga taccgaagac agctcatgtt atatcccgcc gtcaaccacc atcaaacagg

961 attttcgcct gctggggcaa accagcgtgg accgcttgct gcaactctct cagggccagg

1021 cggtgaaggg caatcagctg ttgcccgtct cactggtgaa aagaaaaacc accctggcgc

1081 ccaatacgca aaccgcctct ccccgcgcgt tggccgattc attaatgcag ctggcacgac

1141 aggtttcccg actggaaagc gggcagtgag cgcaacgcaa ttaatgtgag ttagctcact

1201 cattaggcac cccaggcttt acactttatg cttccggctc gtatgttgtg tggaattgtg

1261 agcggataac aatttcacac aggaaacagc tatgaccatg attacgaatt catgagcaat

1321 ctgtcgctcg atttttcgga taatactttt caacctctgg ccgcgcgtat gcggccagaa

1381 aatttagcac agtatatcgg ccagcaacat ttgctggctg cggggaagcc gttgccgcgc

1441 gctatcgaag ccgggcattt acattctatg atcctctggg ggccgccggg taccggcaaa

1501 acaactctcg ctgaagtgat tgcccgctat gcgaacgctg atgtggaacg tatttctgcc

1561 gtcacctctg gcgtgaaaga gattcgcgag gcgatcgagc gcgcccggca aaaccgcaat

1621 gcaggtcgcc gcactattct ttttgttgac gaagttcacc gtttcaacaa aagccagcag

1681 gatgcatttc tgccacatat tgaagacggc accatcactt ttattggcgc aaccactgaa

1741 aacccgtcgt ttgagcttaa ttcggcactg ctttcccgtg cccgtgtcta tctgttgaaa

1801 tccctgagta cagaggatat tgagcaagta ctaactcagg cgatggaaga caaaacccgt

1861 ggctatggtg gtcaggatat tgttctgcca gatgaaacac gacgcgccat tgctgaactg

1921 gtgaatggcg acgcgcgccg ggcgttaaat acgctggaaa tgatggcgga tatggccgaa

1981 gtcgatgata gcggtaagcg ggtcctgaag cctgaattac tgaccgaaat cgccggtgaa

2041 cgtagcgccc gctttgataa caaaggcgat cgcttttacg atctgatttc cgcactgcat

2101 aagtcggtac gtggtagcgc acccgatgcg gcgctgtact ggtatgcgcg aattattacc

2161 gctggtggcg atccgttata tgtcgcgcgt cgctgtctgg cgattgcgtc tgaagacgtc

2221 ggtaatgccg atccacgggc gatgcaggtg gcaattgcgg cctgggattg ctttactcgc

2281 gttggcccgg cggaaggtga acgcgccatt gctcaggcga ttgtttacct ggcctgcgcg

2341 ccaaaaagca acgctgtcta cactgcgttt aaagccgcgc tggccgatgc tcgcgaacgc

2401 ccggattatg acgtgccggt tcatttgcgt aatgcgccga cgaaattaat gaaggaaatg

2461 ggctacgggc aggaatatcg ttacgctcat gatgaagcaa acgcttatgc tgccggtgag

2521 gtttacttcc cgccggaaat agcacaaaca cgctattatt tcccgacaaa caggggcctt

2581 gaaggcaaga ttggcgaaaa gctcgcctgg ctggctgaac aggatcaaaa tagccccata

2641 aaacgctacc gttaaggatc cgtcgacctg cagccaagct tactccccat ccccccgggg

2701 ggcaataagg gctgcacgcg cacttttatc cgcctctgct gcgctccgcc accgtacgta

2761 aatttatggt tggttatgaa atgctggcag agacccagcg agacctgacc gcagaacagg

2821 cagcagagcg tttgcgcgca gtcagcgata tccattttcg cgagtccgga gtgtaagaaa

2881 tgagtctgaa agaaaaaaca caatctctgt ttgccaacgc atttggctac cctgccactc

2941 acaccattca ggcgcctggc cgcgtgaatt tgattggtga acacaccgac tacaacgacg

3001 gtttcgttct gccctgcgcg attgattatc aaaccgtgat cgatcccgtc gttttacaac

3061 gtcgtgactg ggaaaaccct ggcgttaccc aacttaatcg ccttgcagca catccccctt

3121 tcgccagctg gcgtaatagc gaagaggccc gcaccgatcg cccttcccaa cagttgcgca

3181 gcctgaatgg cgaatggcgc tttgcctggt ttccggcacc agaagcggtg ccggaaagct

3241 ggctggagtg cgatcttcct gaggccgata ctgtcgtcgt cccctcaaac tggcagatgc

3301 acggttacga tgcgcccatc tacaccaacg taacctatcc cattacggtc aatccgccgt

3361 ttgttcccac ggagaatccg acgggttgtt actcgctcac atttaatgtt gatgaaagct

3421 ggctacagga aggccagacg cgaattattt ttgatggcgt taactcggcg tttcatctgt

3481 ggtgcaacgg gcgctgggtc ggttacggcc aggacagtcg tttgccgtct gaatttgacc

3541 tgagcgcatt tttacgcgcc ggagaaaacc gcctcgcggt gatggtgctg cgttggagtg

3601 acggcagtta tctggaagat caggatatgt ggcggatgag cggcattttc cgtgacgtct

3661 cgttgctgca taaaccgact acacaaatca gcgatttcca tgttgccact cgctttaatg

3721 atgatttcag ccgcgctgta ctggaggctg aagttcagat gtgcggcgag ttgcgtgact

3781 acctacgggt aacagtttct ttatggcagg gtgaaacgca ggtcgccagc ggcaccgcgc

3841 ctttcggcgg tgaaattatc gatgagcgtg gtggttatgc cgatcgcgtc acactacgtc

3901 tcaacgtcga aaacccgaaa ctgtggagcg ccgaaatccc gaatctctat cgtgcggtgg

3961 ttgaactgca caccggcgac ggcacgctga ttgaagcaga agcctgcgat gtcggtttcc

4021 gcgaggtgcg gattgaaaat ggtctgctgc tgctgaacgg caagccgttg ctgattcgag

4081 gcgttaaccg tcacgagcat catcctctgc atggtcaggt catggatgag cagacgatgg

4141 tgcaggatat cctgctgatg aagcagaaca actttaacgc cgtgcgctgt tcgcattatc

4201 cgaaccatcc gctgtggtac acgctgtgcg accgctacgg cctgtatgtg gtggatgaag

4261 ccaatattga aacccacggc atggtgccaa tgaatcgtct gaccgatgat ccgcgctggc

4321 taccggcgat gagcgaacgc gtaacgcgaa tggtgcagcg cgatcgtaat cacccgagtg

4381 tgatcatctg gtcgctgggg aatgaatcag gccacggcgc taatcacgac gcgctgtatc

4441 gctggatcaa atctgtcgat ccttcccgcc cggtgcagta tgaaggcggc ggagccgaca

4501 ccacggccac cgatattatt tgcccgatgt acgcgcgcgt ggatgaagac cagcccttcc

4561 cggctgtgcc gaaatggtcc atcaaaaaat ggctttcgct acctggagag acgcgcccgc

4621 tgatcctttg cgaatacgcc cacgcgatgg gtaacagtct tggcggtttc gctaaatact

4681 ggcaggcgtt tcgtcagtat ccccgtttac agggcggctt cgtctgggac tgggtggatc

4741 agtcgctgat taaatatgat gaaaacggca acccgtggtc ggcttacggc ggtgattttg

4801 gcgatacgcc gaacgatcgc cagttctgta tgaacggtct ggtctttgcc gaccgcacgc

4861 cgcatccagc gctgacggaa gcaaaacacc agcagcagtt tttccagttc cgtttatccg

4921 ggcaaaccat cgaagtgacc agcgaatacc tgttccgtca tagcgataac gagctcctgc

4981 actggatggt ggcgctggat ggtaagccgc tggcaagcgg tgaagtgcct ctggatgtcg

5041 ctccacaagg taaacagttg attgaactgc ctgaactacc gcagccggag agcgccgggc

5101 aactctggct cacagtacgc gtagtgcaac cgaacgcgac cgcatggtca gaagccgggc

5161 acatcagcgc ctggcagcag tggcgtctgg cggaaaacct cagtgtgacg ctccccgccg

5221 cgtcccacgc catcccgcat ctgaccacca gcgaaatgga tttttgcatc gagctgggta

5281 ataagcgttg gcaatttaac cgccagtcag gctttctttc acagatgtgg attggcgata

5341 aaaaacaact gctgacgccg ctgcgcgatc agttcacccg tgcaccgctg gataacgaca

5401 ttggcgtaag tgaagcgacc cgcattgacc ctaacgcctg ggtcgaacgc tggaaggcgg

5461 cgggccatta ccaggccgaa gcagcgttgt tgcagtgcac ggcagataca cttgctgatg

5521 cggtgctgat tacgaccgct cacgcgtggc agcatcaggg gaaaacctta tttatcagcc

5581 ggaaaaccta ccggattgat ggtagtggtc aaatggcgat taccgttgat gttgaagtgg

5641 cgagcgatac accgcatccg gcgcggattg gcctgaactg ccagctggcg caggtagcag

5701 agcgggtaaa ctggctcgga ttagggccgc aagaaaacta tcccgaccgc cttactgccg

5761 cctgttttga ccgctgggat ctgccattgt cagacatgta taccccgtac gtcttcccga

5821 gcgaaaacgg tctgcgctgc gggacgcgcg aattgaatta tggcccacac cagtggcgcg

5881 gcgacttcca gttcaacatc agccgctaca gtcaacagca actgatggaa accagccatc

5941 gccatctgct gcacgcggaa gaaggcacat ggctgaatat cgacggtttc catatgggga

6001 ttggtggcga cgactcctgg agcccgtcag tatcggcgga attacagctg agcgccggtc

6061 gctaccatta ccagttggtc tggtgtcaaa aataataata accgggcagg ccatgtctgc

6121 ccgtatttcg cgtaaggaaa tccattatgt actatttaaa aaacacaaac ttttggatgt

6181 tcggtttatt ctttttcttt tactttttta tcatgggagc ctacttcccg tttttcccga

6241 tttggctaca tgacatcaac catatcagca aaagtgatac gggtattatt tttgccgcta

6301 tttctctgtt ctcgctatta ttccaaccgc tgtttggtct gctttctgac aaactcgggc

6361 tgcgcaaata cctgctgtgg attattaccg gcatgttagt gatgtttgcg ccgttcttta

6421 tttttatctt cgggccactg ttacaataca acattttagt aggatcgatt gttggtggta

6481 tttatctagg cttttgtttt aacgccggtg cgccagcagt agaggcattt attgagaaag

6541 tcagccgtcg cagtaatttc gaatttggtc gcgcgcggat gtttggctgt gttggctggg

6601 cgctgtgtgc ctcgattgtc ggcatcatgt tcaccatcaa taatcagttt gttttctggc

6661 tgggctctgg ctgtgcactc atcctcgccg ttttactctt tttcgccaaa acggatgcgc

6721 cctcttctgc cacggttgcc aatgcggtag gtgccaacca ttcggcattt agccttaagc

6781 tggcactgga actgttcaga cagccaaaac tgtggttttt gtcactgtat gttattggcg

6841 tttcctgcac ctacgatgtt tttgaccaac agtttgctaa tttctttact tcgttctttg

6901 ctaccggtga acagggtacg cgggtatttg gctacgtaac gacaatgggc gaattactta

6961 acgcctcgat tatgttcttt gcgccactga tcattaatcg catcggtggg aaaaacgccc

7021 tgctgctggc tggcactatt atgtctgtac gtattattgg ctcatcgttc gccacctcag

7081 cgctggaagt ggttattctg aaaacgctgc atatgtttga agtaccgttc ctgctggtgg

7141 gctgctttaa atatattacc agccagtttg aagtgcgttt ttcagcgacg atttatctgg

7201 tctgtttctg cttctttaag caactggcga tgatttttat gtctgtactg gcgggcaata

7261 tgtatgaaag catcggtttc cagggcgctt atctggtgct gggtctggtg gcgctgggct

7321 tcaccttaat ttccgtgttc acgcttagcg gccccggccc gctttccctg ctgcgtcgtc

7381 aggtgaatga agtcgcttaa gcaatcaatg tcggatgcgg cgcgacgctt atccgaccaa

7441 catatcataa cggagtgatc gcattgaaca tgccaatgac cgaaagaata agagcaggca

7501 agctatttac cgatatgtgc gaaggcttac cggaaaaaag acttcgtggg aaaacgttaa

7561 tgtatgagtt taatcactcg catccatcag aagttgaaaa aagagaaagc ctgattaaag

7621 aaatgtttgc cacggtaggg gaaaacgcct gggtagaacc gcctgtctat ttctcttacg

7681 gttccaacat ccatataggc cgcaattttt atgcaaattt caatttaacc attgtcgatg

7741 actacacggt aacaatcggt gataacgtac tgattgcacc caacgttact ctttccgtta

7801 cgggacaccc tgtacaccat gaattgagaa aaaacggcga gatgtactct tttccgataa

7861 cgattggcaa taacgtctgg atcggaagtc atgtggttat taatccaggc gtcaccatcg

7921 gggataattc tgttattggc gcgggtagta tcgtcacaaa agacattcca ccaaacgtcg

7981 tggcggctgg cgttccttgt cgggttattc gcgaaataaa cgaccgggat aagcactatt

8041 atttcaaaga ttataaagtt gaatcgtcag tttaaattat aaaaattgcc tgatacgctg

8101 cgcttatcag gcctacaagt tcagcgatct acattagccg catccggcat gaacaaagcg

8161 caggaacaag cgtcgcatca tgcctctttg acccacagct gcggaaaacg tactggtgca

8221 aaacgcaggg ttatgatcat cagcccaacg acgcacagcg catgaaatgc ccagtccatc

8281 aggtaattgc cgctgatact acgcagcacg ccagaaaacc acggggcaag cccggcgatg

8341 ataaaaccga ttccctgcat aaacgccacc agcttgccag caatagccgg ttgcacagag

8401 tgatcgagcg ccagcagcaa acagagcgga aacgcgccgc ccagacctaa cccacacacc

8461 atcgcccaca ataccggcaa ttgcatcggc agccagataa agccgcagaa ccccaccagt

8521 tgtaacacca gcgccagcat taacagtttg cgccgatcct gatggcgagc catagcaggc

8581 atcagcaaag ctcctgcggc ttgcccaagc gtcatcaatg ccagtaagga accgctgtac

8641 tgcgcgctgg caccaatctc aatatagaaa gcgggtaacc aggcaatcag gctggcgtaa

8701 ccgccgttaa tcagaccgaa gtaaacaccc agcgtccacg cgcggggagt gaataccacg

8761 cgaaccggag tggttgttgt cttgtgggaa gaggcgacct cgcgggcgct ttgccaccac

8821 caggcaaaga gcgcaacaac ggcaggcagc gccaccaggc gagtgtttga taccaggttt

8881 cgctatgttg aactaaccag ggcgttatgg cggcaccaag cccaccgccg cccatcagag

8941 ccgcggacca cagccccatc accagtggcg tgcgctgctg aaaccgccgt ttaatcaccg

9001 aagcatcacc gcctgaatga tgccgatccc caccccacca agcagtgcgc tgctaagcag

9061 cagcgcactt tgcgggtaaa gctcacgcat caatgcaccg acggcaatca gcaacagact

9121 gatggcgaca ctgcgacgtt cgctgacatg ctgatgaagc cagcttccgg ccagcgccag

9181 cccgcccatg gtaaccaccg gcagagcggc ccactgccac ggctcctact gctactcgcg

9241 taacaatcta aagtatgtgc cacggactga cgcaatcgtt aaattgacac tatttgatgg

9301 cgtaatttcg accatccgtg atacattgag gctgttccct gggggtcgtt accttccacg

9361 agcaaaacac gtagcccctt cagagccaga tcctgagcaa gatgaacaga aactgaggtt

9421 ttgtaaacgc cacctttatg ggcagcaacc ccgatcaccg gtggaaatac gtcttcagca

9481 cgtcgcaatc gcgtaccaaa cacatcacgc atatgattaa tttgttcaat tgtataacca

9541 acacgttgct caacccgtcc tcgaatttcc atatccgggt gcggtagtcg ccctgctttc

9601 tcggcatctc tgatagcctg agaagaaacc ccaactaaat ccgctgcttc acctattctc

9661 cagcgccggg ttattttcct cgcttccggg ctgtcatcat taaactgtgc aatggcgata

9721 gccttcgtca tttcatgacc agcgtttatg cactggttaa gtgtttccat gagtttcatt

9781 ctgaacatcc tttaatcatt gctttgcgtt tttttattaa atcttgcaat ttactgcaaa

9841 gcaacaacaa aatcgcaaag tcatcaaaaa accgcaaagt tgtttaaaat aagagcaaca

9901 ctacaaaagg agataagaag agcacatacc tcagtcactt attatcacta gcgctcgccg

9961 cagccgtgta accgagcata gcgagcgaac tggcgaggaa gcaaagaaga actgttctgt

10021 cagatagctc ttacgctcag cgcaagaaga aatatccacc gtgggaaaaa ctccaggtag

10081 aggtacacac gcggatagcc aattcagagt aataaactgt gataatcaac cctcatcaat

10141 gatgacgaac taacccccga tatcaggtca catgacgaag ggaaagagaa ggaaatcaac

10201 tgtgacaaac tgccctcaaa tttggcttcc ttaaaaatta cagttcaaaa agtatgagaa

10261 aatccatgca ggctgaagga aacagcaaaa ctgtgacaaa ttaccctcag taggtcagaa

10321 caaatgtgac gaaccaccct caaatctgtg acagataacc ctcagactat cctgtcgtca

10381 tggaagtgat atcgcggaag gaaaatacga tatgagtcgt ctggcggcct ttctttttct

10441 caatgtatga gaggcgcatt ggagttctgc tgttgatctc attaacacag acctgcagga

10501 agcggcggcg gaagtcaggc atacgctggt aactttgagg cagctggtaa cgctctatga

10561 tccagtcgat tttcagagag acgatgcctg agccatccgg cttacgatac tgacacaggg

10621 attcgtataa acgcatggca tacggattgg tgatttcttt tgtttcacta agccgaaact

10681 gcgtaaaccg gttctgtaac ccgataaaga agggaatgag atatgggttg atatgtacac

10741 tgtaaagccc tctggatgga ctgtgcgcac gtttgataaa ccaaggaaaa gattcatagc

10801 ctttttcatc gccggcatcc tcttcagggc gataaaaaac cacttccttc cccgcgaaac

10861 tcttcaatgc ctgccgtata tccttactgg cttccgcaga ggtcaatccg aatatttcag

10921 catatttagc aacatggatc tcgcagatac cgtcatgttc ctgtagggtg ccatcagatt

10981 ttctgatctg gtcaacgaac agatacagca tacgtttttg atcccgggag agactatatg

11041 ccgcctcagt gaggtcgttt gactggacga ttcgcgggct atttttacgt ttcttgtgat

11101 tgataaccgc tgtttccgcc atgacagatc catgtgaagt gtgacaagtt tttagattgt

11161 cacactaaat aaaaaagagt caataagcag ggataacttt gtgaaaaaac agcttcttct

11221 gagggcaatt tgtcacaggg ttaagggcaa tttgtcacag acaggactgt catttgaggg

11281 tgatttgtca cactgaaagg gcaatttgtc acaacacctt ctctagaacc agcatggata

11341 aaggcctaca aggcgctcta aaaaagaaga tctaaaaact ataaaaaaaa taattataaa

11401 aatatccccg tggataagtg gataacccca agggaagttt tttcaggcat cgtgtgtaag

11461 cagaatatat aagtgctgtt ccctggtgct tcctcgctca ctcgagggct tcgccctgtc

11521 gctcgactgc ggcgagcact actggctgta aaaggacaga ccacatcatg gttctgtgtt

11581 cattaggttg ttctgtccat tgctgacata atccgctcca cttcaacgta acaccgcacg

11641 aagatttcta ttgttcctga aggcatattc aaatcgtttt cgttaccgct tgcaggcatc

11701 atgacagaac actacttcct ataaacgcta cacaggctcc tgagattaat aatgcggatc

11761 tctacgataa tgggagattt tcccgactgt ttcgttcgct tctcagtgga taacagccag

11821 cttctctgtt taacagacaa aaacagcata tccactcagt tccacatttc catataaagg

11881 ccaaggcatt tattctcagg ataattgttt cagcatcgca accgcatcag actccggcat

11941 cgcaaactgc acccggtgcc gggcagccac atccagcgca aaaaccttcg tgtagacttc

12001 cgttgaactg atggacttat gtcccatcag gctttgcaga actttcagcg gtataccggc

12061 atacagcatg tgcatcgcat aggaatggcg gaacgtatgt ggtgtgaccg gaacagagaa

12121 cgtcacaccg tcagcagcag cggcggcaac cgcctcccca atccaggtcc tgaccgttct

12181 gtccgtcact tcccagatcc gcgctttctc tgtccttcct gtgcgacggt tacgccgctc

12241 catgggtatt ttcagtgttg ccaccatcgt ctgcagctgg ctgacgtacc aggagtcaga

12301 gagcggaacc agccggtgag tctgctgacc ggcgggcatt ctccccgccg tcctggcagc

12361 tttttcggtc cgttgtttca gggtcgcaag ctgcacaaac ggatacggag gcgcaagcga

12421 aaaatccccc cgcgtcagcg ccagtgcttc attaatgcgt gctccggtgt tccacagtgt

12481 ggccagcagc atcttgcggt gcagatccgg gacgtaatgg agcagggcac tcacttccgg

12541 agccagcaga tattttggca gttcatcatg gaccatcgac atctggcgaa gtgccagagc

12601 tgccggataa tcaatggcaa caggcagcga tgcaggctgc ccggcagaat acactgccga

12661 ggcgtttccc cctggaagct ccctcgtgcg ctctcctgtt ccgaccctgc cgcttaccgg

12721 atacctgtcc gcctttctcc cttcgggaag cgtggcgctt tctcatagct cacgctgtag

12781 gtatctcagt tcggtgtagg tcgttcgctc caagctgggc tgtgtgcacg aaccccccgt

12841 tcagcccgac cgctgcgcct tatccggtaa ctatcgtctt gagtccaacc cggtaagaca

12901 cgacttatcg ccactggcag cagccactgg taacaggatt agcagagcga ggtatgtagg

12961 cggtgctaca gagttcttga agtggtggcc taactacggc tacactagaa ggacagtatt

13021 tggtatctgc gctctgctga agccagttac cttcggaaaa agagttggta gctcttgatc

13081 cggcaaacaa accaccgctg gtagcggtgg tttttttgtt tgcaagcagc agattacgcg

13141 cagaaaaaaa ggatctcaag aagatccttt gatcttttct acggggtctg acgctcagtg

13201 gaacgaaaac tcacgttaag ggattttggt catgagatta tcaaaaagga tcttcaccta

13261 gatcctttta aattaaaaat gaagttttaa atcaatctaa agtatatatg agtaaacttg

13321 gtctgacagt taccaatgct taatcagtga ggcacctatc tcagcgatct gtctatttcg

13381 ttcatccata gttgcctgac tccccgtcgt gtagataact acgatacggg agggcttacc

13441 atctggcccc agtgctgcaa tgataccgcg agacccacgc tcaccggctc cagatttatc

13501 agcaataaac cagccagccg gaagggccga gcgcagaagt ggtcctgcaa ctttatccgc

13561 ctccatccag tctattaatt gttgccggga agctagagta agtagttcgc cagttaatag

13621 tttgcgcaac gttgttgcca ttgctgcagg catcgtggtg tcacgctcgt cgtttggtat

13681 ggcttcattc agctccggtt cccaacgatc aaggcgagtt acatgatccc ccatgttgtg

13741 caaaaaagcg gttagctcct tcggtcctcc gatcgttgtc agaagtaagt tggccgcagt

13801 gttatcactc atggttatgg cagcactgca taattctctt actgtcatgc catccgtaag

13861 atgcttttct gtgactggtg agtactcaac caagtcattc tgagaatagt gtatgcggcg

13921 accgagttgc tcttgcccgg cgtcaacacg ggataatacc gcgccacata gcagaacttt

13981 aaaagtgctc atcattggaa aacgttcttc ggggcgaaaa ctctcaagga tcttaccgct

14041 gttgagatcc agttcgatgt aacccactcg tgcacccaac tgatcttcag catcttttac

14101 tttcaccagc gtttctgggt gagcaaaaac aggaaggcaa aatgccgcaa aaaagggaat

14161 aagggcgaca cggaaatgtt gaatactcat actcttcctt tttcaatatt attgaagcat

14221 ttatcagggt tattgtctca tgagcggata catatttgaa tgtatttaga aaaataaaca

14281 aataggggtt ccgcgcacat ttccccgaaa agtgccacct gacgtctaag aaaccattat

14341 tatcatgaca ttaacctata aaaataggcg tatcacgagg ccctttcgtc ttcaa

//

**2. pEAW1176 - serS gene in pBAD delta Nco-Nde in NdeI and EcoRI sites**

AAGAAACCAATTGTCCATATTGCATCAGACATTGCCGTCACTGCGTCTTTTACTGGCTCTTCTCGCTAAC

CAAACCGGTAACCCCGCTTATTAAAAGCATTCTGTAACAAAGCGGGACCAAAGCCATGACAAAAACGCGTAACAAAAGTGTCTATAATCACGGCAGAAAAGTCCACATTGATTATTTGCACGGCGTCACACTTTGCTATGCCATAGCATTTTTATCCATAAGATTAGCGGATCCTACCTGACGCTTTTTATCGCAACTCTCTACTGTTTCTCCATACCCGTTTTTTGGGCTAACAGGAGGAATTAAcat

atgctcgatc ccaatctgct gcgtaatgag ccagacgcag tcgctgaaaa

actggcacgc cggggcttta agctggatgt agataagctg ggcgctcttg aagagcgtcg

taaagtattg caggtcaaaa cggaaaacct gcaagcggag cgtaactccc gatcgaaatc

cattggccag gcgaaagcgc gcggggaaga tatcgagcct ttacgtctgg aagtgaacaa

actgggcgaa gagctggatg cagcaaaagc cgagctggat gctttacagg ctgaaattcg

cgatatcgcg ctgaccatcc ctaacctgcc tgcagatgaa gtgccggtag gtaaagacga

aaatgacaac gttgaagtca gccgctgggg taccccgcgt gagtttgact ttgaagttcg

tgaccacgtg acgctgggtg aaatgcactc tggcctcgac tttgcagctg cagttaagct

gactggttcc cgctttgtgg taatgaaagg gcagattgct cgcatgcacc gcgcactgtc

gcagtttatg ctggatctgc ataccgaaca gcatggctac agtgagaact atgttccgta

cctggttaac caggacacgc tgtacggtac gggtcaactg ccgaaatttg ctggcgatct

gttccatact cgtccgctgg aagaagaagc agacaccagt aactatgcgc tgatcccaac

ggcagaagtt ccgctgacta acctggtgcg cggtgaaatc atcgatgaag atgatctgcc

aattaagatg accgcccaca ccccatgctt ccgttctgaa gccggttcat atggtcgtga

cacccgtggt ctgatccgta tgcaccagtt cgacaaagtt gaaatggtgc agatcgtgcg

cccagaagac tcaatggcgg cgctggaaga gatgactggt catgcagaaa aagtcctgca

gttgctgggc ctgccgtacc gtaaaatcat cctttgcact ggcgacatgg gctttggcgc

ttgcaaaact tacgacctgg aagtatggat cccggcacag aacacctacc gtgagatctc

ttcctgctcc aacgtttggg atttccaggc acgtcgtatg caggcacgtt gccgcagcaa

gtcggacaag aaaacccgtc tggttcatac cctgaacggt tctggtctgg ctgttggtcg

tacgctggtt gcagtaatgg aaaactatca gcaggctgat ggtcgtattg aagtaccaga

agttctgcgt ccgtatatga acggactgga atatattggc taa GAATTCGAAGCTTGGGCCCGAACAAAAACTCATCTCAGAAGAGGATCTGAATAGCGCCGTCGA

CCATCATCATCATCATCATTGAGTTTAAACGGTCTCCAGCTTGGCTGTTTTGGCGGATGAGAGAAGATTTTCAGCCTGATACAGATTAAATCAGAACGCAGAAGCGGTCTGATAAAACAGAATTTGCCTGGCGGCAGTAGCGCGGTGGTCCCACCTGACCCCATGCCGAACTCAGAAGTGAAACGCCGTAGCGCCGATGGTAGTGTGGGGTCTCCCCATGCGAGAGTAGGGAACTGCCAGGCATCAAATAAAACGAAAGGCTCAGTCGAAAGACTGGGCCTTTCGTTTTATCTGTTGTTTGTCGGTGAACGCTCTCCTGAGTAGGACAAATCCGCCGGGAGCGGATTTGAACGTTGCGAAGCAACGGCCCGGAGGGTGGCGGGCAGGACGCCCGCCATAAACTGCCAGGCATCAAATTAAGCAGAAGGCCATCCTGACGGATGGCCTTTTTGCGTTTCTACAAACTCTTTTGTTTATTTTTCTAAATACATTCAAATATGTATCCGCTCATGAGACAATAACCCTGATAAATGCTTCAATAATATTGAAAAAGGAAGAGTATGAGTATTCAACATTTCCGTGTCGCCCTTATTCCCTTTTTTGCGGCATTTTGCCTTCCTGTTTTTGCTCACCCAGAAACGCTGGTGAAAGTAAAAGATGCTGAAGATCAGTTGGGTGCACGAGTGGGTTACATCGAACTGGATCTCAACAGCGGTAAGATCCTTGAGAGTTTTCGCCCCGAAGAACGTTTTCCAATGATGAGCACTTTTAAAGTTCTGCTATGTGGCGCGGTATTATCCCGTGTTGACGCCGGGCAAGAGCAACTCGGTCGCCGCATACACTATTCTCAGAATGACTTGGTTGAGTACTCACCAGTCACAGAAAAGCATCTTACGGATGGCATGACAGTAAGAGAATTATGCAGTGCTGCCATAACCATGAGTGATAACACTGCGGCCAACTTACTTCTGACAACGATCGGAGGACCGAAGGAGCTAACCGCTTTTTTGCACAACATGGGGGATCATGTAACTCGCCTTGATCGTTGGGAACCGGAGCTGAATGAAGCCATACCAAACGACGAGCGTGACACCACGATGCCTGTAGCAATGGCAACAACGTTGCGCAAACTATTAACTGGCGAACTACTTACTCTAGCTTCCCGGCAACAATTAATAGACTGGATGGAGGCGGATAAAGTTGCAGGACCACTTCTGCGCTCGGCCCTTCCGGCTGGCTGGTTTATTGCTGATAAATCTGGAGCCGGTGAGCGTGGGTCTCGCGGTATCATTGCAGCACTGGGGCCAGATGGTAAGCCCTCCCGTATCGTAGTTATCTACACGACGGGGAGTCAGGCAACTATGGATGAACGAAATAGACAGATCGCTGAGATAGGTGCCTCACTGATTAAGCATTGGTAACTGTCAGACCAAGTTTACTCATATATACTTTAGATTGATTTAAAACTTCATTTTTAATTTAAAAGGATCTAGGTGAAGATCCTTTTTGATAATCTCATGACCAAAATCCCTTAACGTGAGTTTTCGTTCCACTGAGCGTCAGACCCCGTAGAAAAGATCAAAGGATCTTCTTGAGATCCTTTTTTTCTGCGCGTAATCTGCTGCTTGCAAACAAAAAAACCACCGCTACCAGCGGTGGTTTGTTTGCCGGATCAAGAGCTACCAACTCTTTTTCCGAAGGTAACTGGCTTCAGCAGAGCGCAGATACCAAATACTGTCCTTCTAGTGTAGCCGTAGTTAGGCCACCACTTCAAGAACTCTGTAGCACCGCCTACATACCTCGCTCTGCTAATCCTGTTACCAGTGGCTGCTGCCAGTGGCGATAAGTCGTGTCTTACCGGGTTGGACTCAAGACGATAGTTACCGGATAAGGCGCAGCGGTCGGGCTGAACGGGGGGTTCGTGCACACAGCCCAGCTTGGAGCGAACGACCTACACCGAACTGAGATACCTACAGCGTGAGCTATGAGAAAGCGCCACGCTTCCCGAAGGGAGAAAGGCGGACAGGTATCCGGTAAGCGGCAGGGTCGGAACAGGAGAGCGCACGAGGGAGCTTCCAGGGGGAAACGCCTGGTATCTTTATAGTCCTGTCGGGTTTCGCCACCTCTGACTTGAGCGTCGATTTTTGTGATGCTCGTCAGGGGGGCGGAGCCTATGGAAAAACGCCAGCAACGCGGCCTTTTTACGGTTCCTGGCCTTTTGCTGGCCTTTTGCTCACATGTTCTTTCCTGCGTTATCCCCTGATTCTGTGGATAACCGTATTACCGCCTTTGAGTGAGCTGATACCGCTCGCCGCAGCCGAACGACCGAGCGCAGCGAGTCAGTGAGCGAGGAAGCGGAAGAGCGCCTGATGCGGTATTTTCTCCTTACGCATCTGTGCGGTATTTCACACCGCATAtaTGGTGCACTCTCAGTACAATCTGCTCTGATGCCGCATAGTTAAGCCAGTATACACTCCGCTATCGCTACGTGACTGGGTCATGGCTGCGCCCCGACACCCGCCAACACCCGCTGACGCGCCCTGACGGGCTTGTCTGCTCCCGGCATCCGCTTACAGACAAGCTGTGACCGTCTCCGGGAGCTGCATGTGTCAGAGGTTTTCACCGTCATCACCGAAACGCGCGAGGCAGCAGATCAATTCGCGCGCGAAGGCGAAGCGGCATGCATAATGTGCCTGTCAAATGGACGAAGCAGGGATTCTGCAAACCCTATGCTACTCCGTCAAGCCGTCAATTGTCTGATTCGTTACCAATTATGACAACTTGACGGCTACATCATTCACTTTTTCTTCACAACCGGCACGGAACTCGCTCGGGCTGGCCCCGGTGCATTTTTTAAATACCCGCGAGAAATAGAGTTGATCGTCAAAACCAACATTGCGACCGACGGTGGCGATAGGCATCCGGGTGGTGCTCAAAAGCAGCTTCGCCTGGCTGATACGTTGGTCCTCGCGCCAGCTTAAGACGCTAATCCCTAACTGCTGGCGGAAAAGATGTGACAGACGCGACGGCGACAAGCAAACATGCTGTGCGACGCTGGCGATATCAAAATTGCTGTCTGCCAGGTGATCGCTGATGTACTGACAAGCCTCGCGTACCCGATTATCCATCGGTGGATGGAGCGACTCGTTAATCGCTTCCATGCGCCGCAGTAACAATTGCTCAAGCAGATTTATCGCCAGCAGCTCCGAATAGCGCCCTTCCCCTTGCCCGGCGTTAATGATTTGCCCAAACAGGTCGCTGAAATGCGGCTGGTGCGCTTCATCCGGGCGAAAGAACCCCGTATTGGCAAATATTGACGGCCAGTTAAGCCATTCATGCCAGTAGGCGCGCGGACGAAAGTAAACCCACTGGTGATACCATTCGCGAGCCTCCGGATGACGACCGTAGTGATGAATCTCTCCTGGCGGGAACAGCAAAATATCACCCGGTCGGCAAACAAATTCTCGTCCCTGATTTTTCACCACCCCCTGACCGCGAATGGTGAGATTGAGAATATAACCTTTCATTCCCAGCGGTCGGTCGATAAAAAAATCGAGATAACCGTTGGCCTCAATCGGCGTTAAACCCGCCACCAGATGGGCATTAAACGAGTATCCCGGCAGCAGGGGATCATTTTGCGCTTCAGCCATACTTTTCATACTCCCGCCATTCAGAG

**3. pEAW903 – recN in SuperGlo GFP**

ORIGIN

1 ttcttgaaga cgaaagggcc tcgtgatacg cctattttta taggttaatg tcatgataat

61 aatggtttct tagacgtcag gtggcacttt tcggggaaat gtgcgcggaa cccctatttg

121 tttatttttc taaatacatt caaatatgta tccgctcatg agacaataac cctgataaat

181 gcttcaataa tattgaaaaa ggaagagtat gagtattcaa catttccgtg tcgcccttat

241 tccctttttt gcggcatttt gccttcctgt ttttgctcac ccagaaacgc tggtgaaagt

301 aaaagatgct gaagatcagt tgggtgcacg agtgggttac atcgaactgg atctcaacag

361 cggtaagatc cttgagagtt ttcgccccga agaacgtttt ccaatgatga gcacttttaa

421 agttctgcta tgtggcgcgg tattatcccg tgttgacgcc gggcaagagc aactcggtcg

481 ccgcatacac tattctcaga atgacttggt tgagtactca ccagtcacag aaaagcatct

541 tacggatggc atgacagtaa gagaattatg cagtgctgcc ataaccatga gtgataacac

601 tgcggccaac ttacttctga caacgatcgg aggaccgaag gagctaaccg cttttttgca

661 caacatgggg gatcatgtaa ctcgccttga tcgttgggaa ccggagctga atgaagccat

721 accaaacgac gagcgtgaca ccacgatgcc tgcagcaatg gcaacaacgt tgcgcaaact

781 attaactggc gaactactta ctctagcttc ccggcaacaa ttaatagact ggatggaggc

841 ggataaagtt gcaggaccac ttctgcgctc ggcccttccg gctggctggt ttattgctga

901 taaatctgga gccggtgagc gtgggtctcg cggtatcatt gcagcactgg ggccagatgg

961 taagccctcc cgtatcgtag ttatctacac gacggggagt caggcaacta tggatgaacg

1021 aaatagacag atcgctgaga taggtgcctc actgattaag cattggtaac tgtcagacca

1081 agtttactca tatatacttt agattgattt aaaacttcat ttttaattta aaaggatcta

1141 ggtgaagatc ctttttgata atctcatgac caaaatccct taacgtgagt tttcgttcca

1201 ctgagcgtca gaccccgtag aaaagatcaa aggatcttct tgagatcctt tttttctgcg

1261 cgtaatctgc tgcttgcaaa caaaaaaacc accgctacca gcggtggttt gtttgccgga

1321 tcaagagcta ccaactcttt ttccgaaggt aactggcttc agcagagcgc agataccaaa

1381 tactgtcctt ctagtgtagc cgtagttagg ccaccacttc aagaactctg tagcaccgcc

1441 tacatacctc gctctgctaa tcctgttacc agtggctgct gccagtggcg ataagtcgtg

1501 tcttaccggg ttggactcaa gacgatagtt accggataag gcgcagcggt cgggctgaac

1561 ggggggttcg tgcacacagc ccagcttgga gcgaacgacc tacaccgaac tgagatacct

1621 acagcgtgag ctatgagaaa gcgccacgct tcccgaaggg agaaaggcgg acaggtatcc

1681 ggtaagcggc agggtcggaa caggagagcg cacgagggag cttccagggg gaaacgcctg

1741 gtatctttat agtcctgtcg ggtttcgcca cctctgactt gagcgtcgat ttttgtgatg

1801 ctcgtcaggg gggcggagcc tatggaaaaa cgccagcaac gcggcctttt tacggttcct

1861 ggccttttgc tggccttttg ctcacatgtt ctttcctgcg ttatcccctg attctgtgga

1921 taaccgtatt accgcctttg agtgagctga taccgctcgc cgcagccgaa cgaccgagcg

1981 cagcgagtca gtgagcgagg aagcggaaga gcgcctgatg cggtattttc tccttacgca

2041 tctgtgcggt atttcacacc gcatatatgg tgcactctca gtacaatctg ctctgatgcc

2101 gcatagttaa gccagtatac actccgctat cgctacgtga ctgggtcatg gctgcgcccc

2161 gacacccgcc aacacccgct gacgcgccct gacgggcttg tctgctcccg gcatccgctt

2221 acagacaagc tgtgaccgtc tccgggagct gcatgtgtca gaggttttca ccgtcatcac

2281 cgaaacgcgc gaggcagctg cggtaaagct catcagcgtg gtcgtgaagc gattcacaga

2341 tgtctgcctg ttcatccgcg tccagctcgt tgagtttctc cagaagcgtt aatgtctggc

2401 ttctgataaa gcgggccatg ttaagggcgg ttttttcctg tttggtcact gatgcctccg

2461 tgtaaggggg atttctgttc atgggggtaa tgataccgat gaaacgagag aggatgctca

2521 cgatacgggt tactgatgat gaacatgccc ggttactgga acgttgtgag ggtaaacaac

2581 tggcggtatg gatgcggcgg gaccagagaa aaatcactca gggtcaatgc cagcgcttcg

2641 ttaatacaga tgtaggtgtt ccacagggta gccagcagca tcctgcgatg cagatccgga

2701 acataatggt gcagggcgct gacttccgcg tttccagact ttacgaaaca cggaaaccga

2761 agaccattca tgttgttgct caggtcgcag acgttttgca gcagcagtcg cttcacgttc

2821 gctcgcgtat cggtgattca ttctgctaac cagtaaggca accccgccag cctagccggg

2881 tcctcaacga caggagcacg atcatgcgca cccgtggcca ggacccaacg ctgcccgaga

2941 tgcgccgcgt gcggctgctg gagatggcgg acgcgatgga tatgttctgc caagggttgg

3001 tttgcgcatt cacagttctc cgcaagaatt gattggctcc aattcttgga gtggtgaatc

3061 cgttagcgag gtgccgccgg cttccattca ggtcgaggtg gcccggctcc atgcaccgcg

3121 acgcaacgcg gggaggcaga caaggtatag ggcggcgcct acaatccatg ccaacccgtt

3181 ccatgtgctc gccgaggcgg cataaatcgc cgtgacgatc agcggtccag tgatcgaagt

3241 taggctggta agagccgcga gcgatccttg aagctgtccc tgatggtcgt catctacctg

3301 cctggacagc atggcctgca acgcgggcat cccgatgccg ccggaagcga gaagaatcat

3361 aatggggaag gccatccagc ctcgcgtcgc gaacgccagc aagacgtagc ccagcgcgtc

3421 ggccgccatg ccggcgataa tggcctgctt ctcgccgaaa cgtttggtgg cgggaccagt

3481 gacgaaggct tgagcgaggg cgtgcaagat tccgaatacc gcaagcgaca ggccgatcat

3541 cgtcgcgctc cagcgaaagc ggtcctcgcc gaaaatgacc cagagcgctg ccggcacctg

3601 tcctacgagt tgcatgataa agaagacagt cataagtgcg gcgacgatag tcatgccccg

3661 cgcccaccgg aaggagctga ctgggttgaa ggctctcaag ggcatcggtc gagatcccgg

3721 tgcctaatga gtgagctaac ttacattaat tgcgttgcgc tcactgcccg ctttccagtc

3781 gggaaacctg tcgtgccagc tgcattaatg aatcggccaa cgcgcgggga gaggcggttt

3841 gcgtattggg cgccagggtg gtttttcttt tcaccagtga gacgggcaac agctgattgc

3901 ccttcaccgc ctggccctga gagagttgca gcaagcggtc cacgctggtt tgccccagca

3961 ggcgaaaatc ctgtttgatg gtggttaacg gcgggatata acatgagctg tcttcggtat

4021 cgtcgtatcc cactaccgag atatccgcac caacgcgcag cccggactcg gtaatggcgc

4081 gcattgcgcc cagcgccatc tgatcgttgg caaccagcat cgcagtggga acgatgccct

4141 cattcagcat ttgcatggtt tgttgaaaac cggacatggc actccagtcg ccttcccgtt

4201 ccgctatcgg ctgaatttga ttgcgagtga gatatttatg ccagccagcc agacgcagac

4261 gcgccgagac agaacttaat gggcccgcta acagcgcgat ttgctggtga cccaatgcga

4321 ccagatgctc cacgcccagt cgcgtaccgt cttcatggga gaaaataata ctgttgatgg

4381 gtgtctggtc agagacatca agaaataacg ccggaacatt agtgcaggca gcttccacag

4441 caatggcatc ctggtcatcc agcggatagt taatgatcag cccactgacg cgttgcgcga

4501 gaagattgtg caccgccgct ttacaggctt cgacgccgct tcgttctacc atcgacacca

4561 ccacgctggc acccagttga tcggcgcgag atttaatcgc cgcgacaatt tgcgacggcg

4621 cgtgcagggc cagactggag gtggcaacgc caatcagcaa cgactgtttg cccgccagtt

4681 gttgtgccac gcggttggga atgtaattca gctccgccat cgccgcttcc actttttccc

4741 gcgttttcgc agaaacgtgg ctggcctggt tcaccacgcg ggaaacggtc tgataagaga

4801 caccggcata ctctgcgaca tcgtataacg ttactggttt cacattcacc accctgaatt

4861 gactctcttc cgggcgctat catgccatac cgcgaaaggt tttgcgccat tcgatggtgt

4921 ccgggatctc gacgctctcc cttatgcgac tcctgcatta ggaagcagcc cagtagtagg

4981 ttgaggccgt tgagcaccgc cgccgcaagg aatggtgcat gcaaggagat ggcgcccaac

5041 agtcccccgg ccacggggcc tgccaccata cccacgccga aacaagcgct catgagcccg

5101 aagtggcgag cccgatcttc cccatcggtg atgtcggcga tataggcgcc agcaaccgca

5161 cctgtggcgc cggtgatgcc ggccacgatg cgtccggcgt agaggatcga gatcttgaag

5221 atgtcctgat tcgtcgctgt gattaccatc tgaatctgat tcatccgaaa gattacagtt

5281 atttcaacac attaagcacc aagctcggct ggtcaaaaaa attattctaa ttttacgcca

5341 gcctctttac tgtatataaa accagtttat actgtacaca ataacagtaa tggtttttca

5401 tacaggaaaa cgcatatggc tagcaaagga gaagaactct tcactggagt tgtcccaatt

5461 cttgttgaat tagatggtga tgttaacggc cacaagttct ctgtcagtgg agagggtgaa

5521 ggtgatgcaa catacggaaa acttaccctg aagttcatct gcactactgg caaactgcct

5581 gttccatggc caacactagt cactactctg tgctatggtg ttcaatgctt ttcaagatac

5641 ccggatcata tgaaacggca tgactttttc aagagtgcca tgcccgaagg ttatgtacag

5701 gaaaggacca tcttcttcaa agatgacggc aactacaaga cacgtgctga agtcaagttt

5761 gaaggtgata cccttgttaa tagaatcgag ttaaaaggta ttgacttcaa ggaagatggc

5821 aacattctgg gacacaaatt ggaatacaac tataactcac acaatgtata catcatggca

5881 gacaaacaaa agaatggaat caaagtgaac ttcaagaccc gccacaacat tgaagatgga

5941 agcgttcaac tagcagacca ttatcaacaa aatactccaa ttggcgatgg ccctgtcctt

6001 ttaccagaca accattacct gtccacacaa tctgcccttt cgaaagatcc caacgaaaag

6061 agagaccaca tggtccttct tgagtttgta acagctgctg ggattacaca tggcatggat

6121 gaactgtaca actgaggatc cggctgctaa caaagcccga aaggaagctg agttggctgc

6181 tgccaccgct gagcaataac tagcataacc ccttggggcc tctaaacggg tcttgagggg

6241 ttttttgctg aaaggaggaa ctatatccgg atatcccgca agaggcccgg cagtaccggc

6301 ataaccaagc ctatgcctac agcatccagg gtgacggtgc cgaggatgac gatgagcgca

6361 ttgttagatt tcatacacgg tgcctgactg cgttagcaat ttaactgtga taaactaccg

6421 cattaaagct tatcgatgat aagctgtcaa acatgagaa

//
